# Incidence and risk factors for glaucoma and its clinical, mental health and economic impact in an elderly population: a longitudinal study

**DOI:** 10.1136/bmjopen-2024-096879

**Published:** 2025-06-06

**Authors:** Catherine Jan, Xin Jin, Mengtian Kang, Jiahao Liu, Wenyi Hu, Ruiye Chen, Li Li, Mingguang He, Nathan Congdon

**Affiliations:** 1 Centre for Eye Research Australia, Royal Victorian Eye and Ear Hospital, East Melbourne, Victoria, Australia; 2 Ophthalmology, Department of Surgery, The University of Melbourne, Melbourne, Victoria, Australia; 3 Institute of Population Research, Peking University, Beijing, China; 4 Peking University Library, Peking University, Beijing, China; 5 Beijing Tongren Eye Center, Beijing Tongren Hospital, Capital Medical University, Beijing Key Laboratory of Ophthalmology and Visual Sciences, Beijing, People's Republic of China; 6 School of Optometry, The Hong Kong Polytechnic University, Kowloon, Hong Kong, People's Republic of China; 7 Centre for Public Health, Queen’s University Belfast, Belfast, UK; 8 State Key Laboratory of Ophthalmology, Zhongshan Ophthalmic Center, Sun Yat-Sen University, Guangzhou, China; 9 ORBIS International, New York, New York, USA

**Keywords:** Glaucoma, PUBLIC HEALTH, Risk Factors, China

## Abstract

**Abstract:**

**Objectives:**

To investigate the incidence and determinants of glaucoma in an elderly Chinese population, and clinical, mental health and economic impacts.

**Design:**

This nationally representative, longitudinal study assessed self-reported 6-year (from 2011 to 2018) incident glaucoma diagnosis by a physician and measured biological, clinical and socioeconomical participant characteristics at baseline and endline.

**Setting:**

In the first stage, 150 county-level units from across China were randomly selected with a probability-proportional-to-size sampling technique from a frame containing all county-level units nationwide. The sample was stratified by region and within region by urban district or rural county and per capita gross domestic product. The final sample of 150 counties included 30 out of 31 provinces and autonomous regions in China.

**Participants:**

Consenting, community-dwelling Chinese persons aged 50 years and older.

**Primary and secondary outcome measures:**

Incident glaucoma incidence (primary), factors associated with incident glaucoma (secondary), impact of glaucoma (secondary).

**Results:**

Among 9973 individuals, 3.4% reported a glaucoma diagnosis between 2011 and 2018; Central China had the highest incidence (3.95%) and Eastern China the lowest (2.64%) between 2011 and 2018. Those diagnosed with glaucoma during 2011 and 2018 were of older age (beta coefficient: 0.050, 95% CI: 0.001, 0.001, p<0.001), had higher prevalence of diabetes (beta coefficient: 0.049, 95% CI: 0.028, 0.032, p<0.001), hypertension (beta coefficient: 0.019, 95% CI: 0.006, 0.008, p<0.001), smoking (beta coefficient: 0.029, 95% CI: 0.004, 0.020, p=0.004), alcohol consumption (beta coefficient: 0.026, 95% CI: 0.002, 0.017, p<0.009) and illiteracy (beta coefficient: −0.057, 95% CI: −0.030, –0.015, p<0.001). Logistic regression models showed significant association between incidence of the following characteristics and baseline glaucoma: poor self-reported distance vision (beta coefficient: 1.106, 95% CI: 0.701, 1.511, p<0.001), having hypertension (beta coefficient: 0.545, 95% CI: 0.496, 0.593, p<0.001), having diabetes (beta coefficient: 0.388, 95% CI: 0.326, 0.449, p<0.001), not having obesity (beta coefficient: −0.184, 95% CI: −0.239, –0.129, p<0.001) and lower mean value of health utility score of residents’ quality of life (beta coefficient: −0.040, 95% CI: −0.006, 0.776, p<0.001).

**Conclusions:**

Glaucoma incidence rate varies among geographical regions in China. Several risk factors for incident glaucoma were identified. In addition, glaucoma was found to be associated with multiple physical and psychosocial outcomes. Targeted public health strategies are needed, emphasising early detection and better vision care, to alleviate the burden of glaucoma and improve well-being.

**STRENGTHS AND LIMITATIONS OF THIS STUDY**

- The study used a large, nationally representative sample from most of China’s administrative units, selected using standardised protocols.
- Study personnel were trained uniformly to ensure consistent implementation of procedures.
- The self-reported nature of glaucoma diagnosis may have introduced underreporting or misdiagnosis, though prior evidence suggests reasonable accuracy in self-reports.
- The results may have been influenced by the high proportion (42.2%) of participants who did not attend follow-up examinations or provide information on their glaucoma history.

## Introduction

 Glaucoma, the world’s leading cause of irreversible blindness,^1^ poses a significant public health challenge, especially in a rapidly ageing population such as China’s. In China, the burden of glaucoma is increasingly recognised as a substantial health issue due to its impact on vision-related quality of life and the economy.^2 3^ Despite this recognition, there are no comprehensive nationwide studies exploring the incidence rates and determinants associated with incident glaucoma in mainland China.

China is a large and populous country containing many ethnicities. Understanding the epidemiology of glaucoma incidence and its associated risk factors on a national scale is crucial for developing effective preventive strategies, early detection methods and targeted interventions. Glaucoma incidence studies from mainland China are rare.^4^,^6^ One study reported the 5-year cumulative incidence of primary open-angle glaucoma was 1.3% in Bai Chinese aged 55 years and above,^4^ while another study reported the 5-year cumulative incidence of primary glaucoma was 1.6% in rural northern China among people aged 30 years and older.^5^ Previous localised studies have highlighted specific risk factors contributing to glaucoma incidence in certain regions of China, such as increased age,^4 5^ elevated intraocular pressure,^4 5^ lower educational level^4^ and the presence of myopia,^4^ yet a cohesive and comprehensive nationwide analysis is essential to capture the broader landscape.

The current report aims to address this gap by providing a comprehensive assessment of the national glaucoma incidence rates in China and exploring the determinants, risk factors and impact associated with incident glaucoma across diverse geographical regions and demographic groups. This project employed a national longitudinal survey conducted in 30 out of 31 Chinese provinces and autonomous regions (except Tibet, though the study did include one Tibetan county), representative of the adult population 45 years and older between 2011 and 2018.

The aim of this research is to offer insights into the epidemiology of glaucoma in China, aiding policy-makers, healthcare providers and public health experts in formulating targeted preventive strategies, allocating resources effectively and devising tailored interventions to mitigate the burden of glaucoma and preserve ocular health nationally.

## Methods

This study uses data drawn from the same original longitudinal cohort as our previous publication on cataract surgery incidence,^7^ including the same population selection, study settings and statistical analysis framework. As such, some overlap in the Method sections is present due to the consistent methodology applied across both papers. However, the two studies address entirely distinct research questions: while the previous paper focused on the incidence and determinants of cataract surgery uptake—a healthcare service intervention—this manuscript investigates the incidence of glaucoma, a different disease entity and explores its associated clinical, psychosocial and economic consequences.

### Study population

The China Health and Retirement Longitudinal Study (CHARLS) is a nationally representative longitudinal survey among Chinese persons aged 45 years and older (note for this particular paper, we used data from people aged 50 years and above because glaucoma is an age-related disease and this is the most common threshold reported by literature), that includes assessments of biological, social and economic conditions. We used data from the baseline in-person study conducted in 2011 (‘wave 1’) and an in-person follow-up conducted in 2018 (‘wave 4’). Main respondents and spouses in the baseline survey are followed throughout the life of CHARLS, or until they die.^8^ Detailed information on the methodology of CHARLS has been provided elsewhere.^8^ Among 17 250 eligible participants at baseline who provided information on glaucoma status and had not been diagnosed with glaucoma in 2011, 9973 (57.8%) attended the 7-year follow-up and provided glaucoma information in 2018. The identical protocols for collection of the data used in this paper were followed during the baseline and follow-up studies.

The participant cohort was chosen through multistage probability sampling. In the first stage, 150 county-level units were randomly selected with a probability-proportional-to-size (PPS) sampling technique from a frame containing all county-level units in the country, with the exception of the Tibet Autonomous Region. The sample was stratified by region and within region by urban district or rural county and per capita gross domestic product (GDP). The final sample of 150 counties included 28 of China’s 31 administrative units (provinces and autonomous regions). Our sample used the lowest level of government organisation, consisting of administrative villages (cun) in rural areas and neighbourhood*s* (shequ or juweihui) in urban areas, as primary sampling units (PSUs). We selected three PSUs within each county-level unit, using PPS sampling (more details are reported elsewhere).^8^

### Variables

Glaucoma incidence in this paper refers to the proportion of the cohort population aged 50 years and older who reported having a glaucoma diagnosis by a physician in at least one eye between 2011 and 2018, among those who had not been diagnosed in either eye at baseline. This was determined by participants’ response to the question “Have you been diagnosed with glaucoma by a physician before?”, comparing answers between the previous and present round. Age and gender were self-reported variables taken from the baseline 2011 survey.

Rural or urban place of residence was obtained from self-reported hukou. Hukou is a Chinese internal passport system, designed to regulate the movement of rural residents into urban areas; migrants with a rural hukou do not have access to local healthcare as do local urban residents. Hukou also determines the type of public health insurance a person has access to.

Three comorbidities linked with glaucoma were extracted from the CHARLS dataset. Hypertension was defined as either self-report of a physician’s diagnosis, or presence of objective biomarkers (systolic pressure ≥140 and/or diastolic pressure ≥90 mm Hg) during direct physical examination. Diabetes was also defined by self-report of prior diagnosis by a doctor or the presence of a biomarker (hemoglobin A1c, also known as glycated hemoglobin; HbA1c value ≥6.5%) on examination. Depression symptoms were measured as a continuous variable, using the Centre for Epidemiologic Studies Depression Scale-10,^9^ with total score ranging from 0 (minimal symptoms) to 30 (most symptomatic). Other covariates included in this study are household expenditure, representative of economic status, and EuroQol five-dimension questionnaire or EQ-5D health utility scores, an indicator of quality of life, and level of attained education.

### Patient and public involvement

None.

### Statistical analysis

All statistical analyses were performed using Stata V.14.0 (StataCorp). The t-test was used to compare continuous variables, while Pearson χ^2^ or Fisher’s exact tests were used for the comparison of categorical data. To assess the accuracy of household expenditure data from the sample as an index of income in the province, a scatterplot graph was made comparing household expenditure to provincial GDP data. The 7-year incidence of newly diagnosed glaucoma stratified by age and gender was calculated. Age was defined in our analysis as the age at baseline. Univariate and multivariate logistic regression models were used to test for associations with incident glaucoma over the 7-year follow-up period. We ran regression analyses including the whole sample of eligible participants, aged 50 years and above. Variables in univariate models with p<0.05 were entered into the multivariate models. In addition, univariate logistic regression models were applied to investigate the association of baseline (2011) glaucoma with subsequent (2018) social and economic variables. We applied sample weights to the glaucoma incidence between 2011 and 2018. These weights were constructed from weights for the structure of age and gender from the baseline wave of the CHARLS study. A p<0.05 was defined as statistically significant.

## Results

Among 9973 eligible participants aged 50 years and above (table 1), 342 (3.4%) self-reported glaucoma diagnosis by a physician, while 9631 (96.6%) did not. Those who were diagnosed with glaucoma were significantly older (62.9±7.99 vs 60.8±7.49, p<0.001), were more likely to be female (60.8% vs 51.0%, p<0.001), to self-report poor distance vision at baseline (38.6% vs 22.1%, p<0.001), to have diabetes (14.3% vs 7.6%, p<0.001), to report currently drinking alcohol and drinking in the past year (64.9% vs 57.8%, p<0.05), currently smoking and smoking in the past (75.2% vs 67.4%, p=0.01), to be illiterate (45.3% vs 30.4%, p<0.001), have a lower health utility quality of life score (0.72±0.23 vs 0.79±0.25, p<0.001), higher depression score (10.5±7.08 vs 8.49±6.32, p<0.001 and to be unmarried (23.1% vs 18.3%) (table 1). The proportion of persons living in the different geographical regions of China also differed between those with versus without glaucoma (Eastern region 26.9% vs 35.2%, Central region 37.7% vs 32.6% and Western region 35.4% vs 32.3%, p<0.01).

**Table 1:** Baseline characteristics of 9973 participants aged 50 and above who participated in both the baseline and follow-up surveys

Baseline characteristic	Glaucoma	Non-glaucoma	P value
N	342	9631	
Mean age, years (SD)	62.9 (7.99）	60.81 (7.49）	**<0.001**
Self-rated distance Vision, n (%)			
Poor	132 (38.6)	2125 (22.06)	**<0.001**
Excellent, very good, good or fair	190 (55.6)	6956 (72.23)	
Hypertension present, n (%)	157 (45.9)	3978 (41.3)	0.232
Diabetes present, n (%)	49 (14.3)	731 (7.59)	**<0.001**
Obesity present, n (%)	83 (24.27)	2334 (24.23)	0.512
Male gender, n (%)	134 (39.2)	4714 (49.0)	**<0.001**
Drinking alcohol currently and also drinking in the past year	222 (64.9)	5565 (57.8)	**0.029**
Currently smoking and also smoking in the past	257 (75.2)	6488 (67.4)	**0.010**
Literacy, n (%)			
Illiterate	155 (45.3)	2932 (30.4)	**<0.001**
EQ-5D score, mean (SD)	0.72 (0.23)	0.79 (0.25)	**<0.001**
Depression score, mean (SD)	10.5 (7.08)	8.49 (6.32)	**<0.001**
Rural hukou, n (%)	279 (81.6)	7728 (80.2)	0.817
Mean log household per capita expenditure in USD, mean (SD)	4.59 (0.88)	4.62 (0.88)	0.539
Reporting having health insurance, n (%)	323 (94.4)	9057 (94.0)	0.880
Geographical location, n (%)			
Eastern region	92 (26.9)	3387 (35.2)	**0.006**
Central region	129 (37.7)	3138 (32.6)	
Western region	121 (35.4)	3106 (32.3)	
Married and living together	263 (76.9)	7870 (81.7)	**0.024**

A p<0.05 was defined as statistically significant.

EQ-5D, EuroQol five-dimension questionnaire; SD, standard deviation.

Self-reported 7-year glaucoma incidence was 2.67% among the cohort aged 50–59 years, 3.76% at 60–69 years, 5.35% for 70–79 years and 4.79% for 80+ years (table 2).

**Table 2:** Glaucoma incidence of total population and total self-reported glaucoma between 2011 and 2018, stratified by age group

Age group (years)	Cases	Glaucoma incidence, among entire population (%)	Among those with self-reported incident glaucoma (%)
50–59	134	2.67	39.18
60–69	132	3.76	38.6
70–79	67	5.35	19.59
80+	9	4.79	2.63
Total	342	3.43	100

Table 3 shows the glaucoma incidence between 2011 and 2018 stratified by geographic region and province. Central China had the highest incidence (3.95%) and Eastern China the lowest (2.64%).

**Table 3:** Glaucoma incidence between 2011 and 2018, stratified by geographical region and province

Province	Self-reported glaucoma incidence, n (%)
Eastern China	
Tianjin	1 (1.30)
Beijing	1 (3.03)
Shanghai	1 (4.17)
Jiangsu	16 (3.16)
Zhejiang	11 (2.69)
Guangdong	8 (1.61%)
Liaoning	13 (4.38)
Fujian	7 (2.49)
Shandong	13 (1.48)
Jilin	13 (5.78)
Hebei	21 (4.45)
Heilongjiang	5 (3.29)
Total	92 (2.64)
Central China	
Hubei	7 (2.22)
Shanxi	11 (3.44)
Hunan	13 (2.80)
Henan	34 (4.32)
Jiangxi	15 (2.92)
Anhui	31 (6.34)
Total	129 (3.95)
Western China	
Inner Mongolia	19 (4.39)
Chongqing	15 (2.92)
Shaanxi	11 (3.56)
Xinjiang	3 (7.14)
Qinghai	4 (4.88)
Sichuan	40 (4.38)
Guangxi	8 (2.22)
Gansu	8 (3.56)
Yunnan	15 (2.55)
Guizhou	3 (2.44)
TOTAL	121 (3.75)

In regression models of potential predictors of incidence of glaucoma among persons aged 50 years and above at baseline, older age (beta coefficient: 0.050, 95% CI: 0.001, 0.001, p<0.001), having hypertension (beta coefficient: 0.019, 95% CI: 0.006, 0.008, p<0.001) or diabetes (beta coefficient: 0.049, 95% CI: 0.028, 0.032, p<0.001), being female (beta coefficient: 0.036, 95% CI: 0.006, 0.020, p<0.001), recent smoking (beta coefficient: 0.029, 95% CI: 0.004, 0.020, p=0.004) and consumption of alcohol (beta coefficient: 0.026, 95% CI: 0.002, 0.017, p<0.009) and illiteracy (beta coefficient: −0.057, 95% CI: −0.030 to –0.015, p<0.001), were all significantly associated with increased incidence in the univariate model, and all factors except female sex remained significant in the multivariate model (table 4).

**Table 4:** Regression models of potential predictors of self-reported incident glaucoma diagnosed by any physician between 2011 and 2018 among a nationally representative sample of Chinese persons aged ≥50 years who did not have glaucoma at baseline (n=9973)

Baseline characteristic	Univariate model		Multivariate model	
	β (95% CI)	P value	β (95% CI)	P value
Age, years	0.050 (0.001, 0.002)	**<0.001**	0.035 (0.001, 0.001)	**<0.001**
Hypertension present (adjusted for age)	0.019 (0.006, 0.008)	**<0.001**	0.005 (0.001, 0.003)	**0.001**
Diabetes present (adjusted for age)	0.049 (0.028, 0.032)	**<0.001**	0.052 (0.030, 0.034)	**<0.001**
Female	0.036 (0.006, 0.020)	**<0.001**	−0.001 (−0.002, 0.001)	0.611
Currently smoking and also smoking in the past	0.029 (0.004, 0.020)	**0.004**	0.020 (0.007, 0.010)	**<0.001**
Drinking alcohol currently and also drinking in the past year	0.026 (0.002, 0.017)	**0.009**	0.013 (0.004, 0.006)	**<0.001**
Literate	−0.057 (−0.030, 0.015)	**<0.001**	−0.044 (−0.019, 0.016)	**<0.001**
Rural (vs non-rural) hukou	0.006 (−0.006, 0.012)	0.544		

Bold type indicates values significant at p<0.05.

Incidence of glaucoma is 3.43% in the general study population, 3.94% among those with baseline hypertension, 6.52% among those with diabetes, 3.53% among those with obesity, 11.54% among those with low quality of life, 4.40% among those with depression and 3.35% among those with below average household per capita expenditure.

Logistic regression models showed significant association between incidence of the following characteristics and baseline glaucoma: poor self-reported distance vision (beta coefficient: 1.106, 95% CI: 0.701, 1.511, p<0.001), having hypertension (beta coefficient: 0.545, 95% CI: 0.496, 0.593, p<0.001), having diabetes (beta coefficient: 0.388, 95% CI: 0.326, 0.449, p<0.001), not having obesity (beta coefficient: −0.184, 95% CI: −0.239, –0.129, p<0.001) and lower mean value of health utility score of residents’ quality of life (beta coefficient: −0.040, 95% CI: −0.006, 0.776, p<0.001) (table 5).

**Table 5:** Logistic regression models of potential subsequent clinical, mental health and socioeconomic associations with baseline (2011) self-reported glaucoma diagnosed by any physician, among a nationally representative sample of Chinese persons aged ≥50 years (n=9973)

2018 status	β (95% CI)	P value
Poor self-reported distance vision	1.106(0.701, 1.511)	**<0.001**
Hypertension present (adjusted for age)	0.545(0.496, 0.593)	**<0.001**
Diabetes present (adjusted for age)	0.388(0.326, 0.449)	**<0.001**
Obesity 2015 (adjusted for 2011 age)*	−0.184(−0.239, 0.129)	**<0.001**
Mean value of health utility score of residents’ quality of life (EQ-5D)	−0.040(−0.134, 0.045)	**<0.001**
Mean depression score (adjusted for age)	0.018(−0.006, 0.776)	0.051
Logarithm of household per capita expenditure	−0.012(−0.336, 0.072)	0.204

Bold type indicates values significant at p<0.05) (Independent variable=2011 glaucoma status, dependent variable=subsequent 2018 clinical, mental health and socioeconomic associations.

* Wave 4 (2018) did not undergo physical examinations or blood tests; therefore, the BMI is based on the 2015 data.

BMI, body mass index; CI, confidence interval; EQ-5D, EuroQol five-dimension questionnaire.

## Discussion

Glaucoma poses a substantial public health challenge globally, especially in ageing populations. A previous study reported the prevalence of glaucoma in the same population; however, glaucoma incidence has not been reported in this population.^10^ Our study aimed to comprehensively assess nationwide glaucoma incidence rates and associated determinants among older Chinese persons, addressing a critical gap in understanding this condition’s epidemiology.

Using data from the CHARLS from 2011 to 2018, we identified significant factors associated with incident glaucoma among adults aged 50 and older. Our findings demonstrated a self-reported glaucoma incidence of 3.4%, revealing demographic, socioeconomic and health-related disparities among those diagnosed with glaucoma versus those without. Affected individuals were older, more likely to report poor vision and exhibit comorbidities like diabetes and experienced lower quality of life scores and higher depression levels.

Regional variations were evident in glaucoma incidence, with Central China reporting the highest incidence rates. Glaucoma development can be attributed to a complex interplay of genetic, environmental and socioeconomic factors.^11^ In Central China, for instance, neovascularisation and trauma were identified as leading causes for secondary glaucoma.^12^ This elevated rate of trauma-related glaucoma may be linked to specific regional activities or occupational hazards prevalent in Central China.^12^ Our findings showed that older age, hypertension, diabetes, illiteracy, smoking and alcohol consumption were associated with increased glaucoma incidence, highlighting the multifactorial nature of its risk factors. Our longitudinal findings are consistent with the cross-sectional baseline paper^10^ showing associations between glaucoma and hypertension, diabetes, smoking and alcohol use. Interestingly, our study found no association between incident glaucoma and socioeconomic or urban–rural status, despite a significant link between illiteracy and incident glaucoma. This contrasts with previous prevalence studies that reported an association between lower socioeconomic status and higher glaucoma prevalence.^13^ These findings highlight the need for more longitudinal studies on glaucoma incidence and risk factors, beyond cross-sectional prevalence studies.

Importantly, our study also underscored the impact of incident glaucoma on individuals’ subsequent health and socioeconomic status. Those diagnosed with glaucoma at baseline showed increased incident comorbidities, lower quality of life scores and financial vulnerabilities, reflecting the broader repercussions of this condition beyond vision impairment. These findings not only highlight the importance of early prevention of risk factors, but also emphasise the full-cycle management of glaucoma in alleviating the associated disease burden in terms of health system and society.

Our findings align with previous localised studies in China, highlighting the significance of older age and lower education level as key risk factors for glaucoma.^4 5^ However, our nationwide scope underscores the need for targeted interventions at the national level addressing these risk factors.

The strengths of this study include use of a large, nationally representative sample, drawn from most of China’s administrative units and selected using standardised protocols. Study personnel were trained in standard fashion to ensure all procedures were carried out as described. Our findings provide a basis for comparison with other low-income and middle-income countries and especially those undergoing rapid economic transitions. Limitations include the self-reported nature of glaucoma diagnosis and potential under-reporting or misdiagnosis, though evidence has suggested that self-reported medical diagnosis is reasonably accurate. A study showed that of the 200 subjects with medical record information indicating glaucoma, 165 (77.0%) correctly self-reported their glaucoma diagnosis.^14^ Among the 130 subjects with medical record information indicating glaucoma suspect or ocular hypertension, 109 (83.9%) correctly self-reported no glaucoma diagnosis.^14^ However, further studies validating self-reported diagnoses against medical records are warranted. An additional limitation is that the results may have been influenced by patients (42.2%) who did not attend the follow-up examination, or who failed to provide information on their glaucoma history. This might induce attrition bias in the results.

## Conclusions

Our study provides critical insights into glaucoma incidence rates, risk factors and their association with long-term disease and psychosocial outcomes on the Chinese population. These findings underscore the need for national-level public health strategies focusing on early detection, improved vision care and targeted interventions to mitigate the burden of glaucoma and enhance overall well-being. This research lays the foundation for further investigations and policy formulations aimed at addressing the multifaceted challenges posed by glaucoma in China, ultimately contributing to enhanced eye health and improved quality of life.

## Data Availability

Data are available in a public, open access repository.
