# Supplementary material for: Incidence and risk factors for glaucoma and its clinical, mental health and economic impact in an elderly population: a longitudinal study
Source: BMJ Open. 2025 Jun 6;15(6):e096879. doi: 10.1136/bmjopen-2024-096879 (PMC12161292; doi:10.1136/bmjopen-2024-096879)
Supplement: Supplementary file 1 [file bmjopen-2024-096879.reviewer_comments.pdf]

PEER REVIEW HISTORY

BMJ Open publishes all reviews undertaken for accepted manuscripts. Reviewers are asked to complete a checklist review form and are provided with free text boxes to elaborate on their assessment. These free text comments are reproduced below.

ARTICLE DETAILS

Title (Provisional)

Incidence and risk factors for glaucoma, and its clinical, mental health, and economic impact in an elderly population: a longitudinal study

Authors

Jan, Catherine; Jin, Xin; Kang, Mengtian; Liu, Jiahao; Hu, Wenyi; Chen, Ruiye; Li, Li; He, Mingguang; Congdon, Nathan

---

VERSION 1 - REVIEW

**Reviewer**	**1**
**Name**	**Dayal, Ashutosh**
**Affiliation**	**PBMA'S HV Desai Eye Hospital**
**Date**	**21-Jan-2025**
**COI**	**None**

At the onset, I would like to congratulate the authors for undertaking this important study. Glaucoma has a very high global morbidity burden and can affect quality of life in multiple ways.

However I would like clarification/ modification on a certain points:

1. The reference list is incomplete. Please provide complete list of references.

2. Patient drop out (lost to follow up) from the baseline is high (almost 42%). This might induce attrition bias in the results.

3. Would the authors like to elaborate upon the probable causes of disparity in incidence of glaucoma in various geographic regions? (eg ethnicity, lifestyle habits, etc)

4. Authors have mentioned "A study showed that of the 200 subjects with medical record information indicating glaucoma, 165 (77.0%) correctly self-reported their glaucoma diagnosis". None of the study cited by the authors have >95% accuracy in self reporting of glaucoma when matched with health records. This might induce a bias. Ideally medical records should have been tallied for a proportion of participants.

5. Table 1 mentions illiteracy and depression to be more prevalent in self reported glaucoma group. However, illiteracy, itself per se, can lead to poor socioeconomic status and hence affect mental health. Was the confounding effect of illiteracy/socioeconomic status of depression scores taken into consideration?

**Reviewer**	**2**
**Name**	**Ogbonna, Grace**
**Affiliation**	**Department of Optometry, Mzuzu University, Mzuzu University**
**Date**	**25-Jan-2025**
**COI**	**None**

This is a unique and interesting work, however, the discussion seems to be very brief and does not talk about the major study variables, what the study found, how it compared to other studies, and its significance. Similarly, the age of the participants seems to have been confused across the document.

Topic should reflect the study location e.g elderly population in china

Abstract (Mino revisions)

1. The objective is missing its before clinical, mental health and economic impact
2. Duration of the study e.g 2011 to 2018 seems to me 7 years instead of 6. If the duration was not up to 7 years you may wish to say 6 years and how many moths the study lasted for.
3. What was the study approach?
4. People who are 50 to 64 are not elderly, except if this is a nationally specific definition of elderly
5. How were the results analyzed?
6. What was used for data collection?
7. Page 2 line 26 indicates that the study participants were 50 years and older contradicting what was written on line 36 page 4.
8. The statement on lines 57-58 page 2 “In addition, glaucoma was found to be associated with multiple physical and psychosocial outcomes” is not reflected in the result in the abstract. While quality of life was mentioned, it didn’t reflect psychosocial impact.

Introduction

1. Please replace was with as in line 19 e.g. ...primary open angle glaucoma (POAG) was 1.3% in Bai Chinese aged 55 years and above. Please do the same
2. Line 40 of page 4 is about the significance of the study not necessarily the aim of the study.

Methodology

1. Please can the method clearly show the study setting and location? Although the statement "The China Health and Retirement Longitudinal Study (CHARLS)" implies that the study was done in China, it would be good for early researchers whose English mastery may not be very sophisticated to read this document and clearly know where the study was done.
2. Please recast the sentence on Page 5 line 5-6 for clarity.

Analysis

1. Page 6 line 17, how was this arrived at? Does it mean that household expenditure did not change in 7 years r was the last year used for this?
2. How many contacts did the researchers have with the participants?
3. Please can the study be clear of the age at base line. Initially, it was 45 and above, but over time, the document asserted 50 years.

Discussion

The discussion is rather sparse and does not delve into the findings, their significance and how it compared to other studies across the globe. This would be very helpful

Limitations of the study:

1. Because people self-reported, there is a possibility of recall bias as such patients' age could be wrongly reported.
2. It is also possible that participant' social economic status changed along the line and this may not have been accounted for in the study.

VERSION 1 - AUTHOR RESPONSE

Reviewer: 1
Dr. Ashutosh Dayal, PBMA'S HV Desai Eye Hospital
Comments to the Author:
At the onset, I would like to congratulate the authors for undertaking this important study. Glaucoma has a very high global morbidity burden and can affect quality of life in multiple ways.

Response:

Thank you for your acknowledgment.

However I would like clarification/ modification on a certain points:
1. The reference list is incomplete. Please provide complete list of references.

Response:
We have reviewed and updated the reference list to ensure it is complete and follows the journal's format.

2. Patient drop out (lost to follow up) from the baseline is high (almost 42%). This might induce attrition bias in the results.

Response:
We acknowledge this limitation and have included in the **Limitations** section discussing the potential impact of attrition bias.

Revised text:

An additional limitation is that the results may have been influenced by patients (42.2%) who did not attend the follow-up examination, or who failed to provide information on their glaucoma history. This might induce attrition bias in the results.

3. Would the authors like to elaborate upon the probable causes of disparity in incidence of glaucoma in various geographic regions? (eg ethnicity, lifestyle habits, etc)

Response:
We have expanded the **Discussion** section to explore potential factors contributing to regional disparities in glaucoma incidence. Relevant references have been added to support this discussion.

Revised text:

"Regional variations were evident in glaucoma incidence, with Central China reporting the highest incidence rates. Glaucoma development can be attributed to a complex interplay of genetic, environmental, and socioeconomic factors.[10] In Central China, for instance, neovascularisation and trauma were identified as leading causes for secondary glaucoma.[11] This elevated rate of trauma-related glaucoma may be linked to specific regional activities or occupational hazards prevalent in Central China.[11] Our findings showed that older age, hypertension, diabetes, illiteracy, smoking, and alcohol consumption were associated with increased glaucoma incidence, highlighting the multifactorial nature of its risk factors. Our longitudinal findings are consistent with the cross-sectional baseline paper[9] showing associations between glaucoma and hypertension, diabetes, smoking, and alcohol use."

4. Authors have mentioned "A study showed that of the 200 subjects with medical record information indicating glaucoma, 165 (77.0%) correctly self-reported their glaucoma diagnosis". None of the study cited by the authors have >95% accuracy in self reporting of glaucoma when matched with health records. This might induce a bias. Ideally medical records should have been tallied for a proportion of participants.

Response:
We agree with this concern. We have revised the **Discussion** section to clarify the potential limitations of using self-reported data in the **strengths and limitations** paragraph. We also

highlighted the need for further studies validating self-reported diagnoses against medical records.

Revised text:

"Limitations include the self-reported nature of glaucoma diagnosis and potential underreporting or misdiagnosis, though though evidence has suggested that self-reported medical diagnosis is reasonably accurate. A study showed that of the 200 subjects with medical record information indicating glaucoma, 165 (77.0%) correctly self-reported their glaucoma diagnosis.[12] Among the 130 subjects with medical record information indicating glaucoma suspect or ocular hypertension, 109 (83.9%) correctly self-reported no glaucoma diagnosis.[12] However, further studies validating self-reported diagnoses against medical records are warranted."

5. Table 1 mentions illiteracy and depression to be more prevalent in self reported glaucoma group. However, illiteracy, itself per se, can lead to poor socioeconomic status and hence affect mental health. Was the confounding effect of illiteracy/socioeconomic status of depression scores taken into consideration?

Response:
Thank you for this valuable observation. We have updated the **Discussion** section to clarify the association between illiteracy, socioeconomic status and self-reported glaucoma. Table 1 showed cross-sectional baseline characteristics and were not the main focus of this study. This longitudinal study found no significant association between socioeconomic status and glaucoma incidence, a discrepancy between cross-sectional studies and longitudinal studies. We also found no significant association between baseline glaucoma and subsequent depression development. More incidence studies are needed to compare risk factors.

Revised text:

"Interestingly, our study found no association between incident glaucoma and socioeconomic or urban-rural status, despite a significant link between illiteracy and incident glaucoma. This contrasts with previous prevalence studies that reported an association between lower socioeconomic status and higher glaucoma prevalence.[12] These findings highlight the need for more longitudinal studies on glaucoma incidence and risk factors, beyond cross-sectional prevalence studies."

Reviewer: 2
Dr. Grace Ogbonna, Department of Optometry, Mzuzu University
Comments to the Author:
This is a unique and interesting work, however, the discussion seems to be very brief and does not talk about the major study variables, what the study found, how it compared to other studies, and its significance.

Response:
As per our answers to reviewer 1, we have significantly expanded the **Discussion** section to more comprehensively address the major findings of our study, compare them with existing literature, and discuss their implications in greater detail.

Similarly, the age of the participants seems to have been confused across the document.

Response:
We have reviewed the manuscript thoroughly and clarified the age ranges of participants consistently across all sections. Age-specific data and analyses have been clearly stated to eliminate any confusion.

Revised text:

"The China Health and Retirement Longitudinal Study (CHARLS) is a nationally-representative longitudinal survey among Chinese persons aged 45 years and older (note for this particular paper, we used data from people aged 50 years and above because glaucoma is an age-related disease and this is the most common threshold reported by literature)…"

"Glaucoma incidence in this paper refers to the proportion of the cohort population aged 50 years and older who reported having glaucoma diagnosis by a physician in at least one eye between 2011 and 2018, among those who had not been diagnosed in either eye at baseline."

Conclusion

We appreciate the constructive feedback provided by the Editor and Reviewers, which has significantly improved our manuscript. **Thank you very much for your time and consideration.**
